# Galloway-Mowat syndrome in Taiwan: *OSGEP* mutation and unique clinical phenotype

**DOI:** 10.1186/s13023-018-0961-9

**Published:** 2018-12-17

**Authors:** Pei-Yi Lin, Min-Hua Tseng, Martin Zenker, Jia Rao, Friedhelm Hildebrandt, Shih-Hua Lin, Chun-Chen Lin, Jui-Hsing Chang, Chyong-Hsin Hsu, Ming-Dar Lee, Shuan-Pei Lin, Jeng-Daw Tsai

**Affiliations:** 1 Department of Pediatrics, MacKay Children’s Hospital, No. 92, Sec. 2, Chung-Shan North Road, Taipei, Taiwan; 2 Department of Pediatrics, Shuang Ho Hospital, Taipei Medical University, New Taipei City, Taiwan; 3 Graduate Institute of Biomedical Informatics, College of Medicine Science and Technology, Taipei Medical University, Taipei, Taiwan; 4 Division of Pediatric Nephrology, Department of Pediatrics, Chang Gung Memorial Hospital, Chang Gung University, Taoyuan, Taiwan; 5 Institute of Human Genetics, University Hospital Magdeburg, Magdeburg, Germany; 6 Department of Medicine, Boston Children’s Hospital, Harvard Medical School, Boston, MA USA; 7 Division of Nephrology, Department of Medicine, Tri-Service General Hospital, National Defense Medical Center, Taipei, Taiwan; 8 Department of Medicine, MacKay Medical College, New Taipei City, Taiwan; 9 MacKay Junior College of Medicine, Nursing and Management, Taipei, Taiwan; 10 Department of Pediatrics, Hsinchu MacKay Memorial Hospital, Hsinchu City, Taiwan; 11 Department of Pediatric Genetics, MacKay Children’s Hospital, Taipei, Taiwan; 12 Department of Pediatrics, Taipei Medical University Hospital, Taipei, Taiwan; 13 Department of Pediatrics, School of Medicine, College of Medicine, Taipei Medical University, Taipei, Taiwan

**Keywords:** Galloway-Mowat syndrome, *OSGEP*, KEOPS, Nephrotic syndrome, Microcephaly, Pachygyria, Hypomyelination, Arachnodactyly, Camptodactyly, Prenatal findings

## Abstract

**Background:**

Galloway-Mowat syndrome (GAMOS) is a rare autosomal recessive disease characterized by the combination of glomerulopathy with early-onset nephrotic syndrome and microcephaly with central nervous system anomalies. Given its clinical heterogeneity, GAMOS is believed to be a genetically heterogenous group of disorders. Recently, it has been reported that mutations in KEOPS-encoding genes, including the *OSGEP* gene, were responsible for GAMOS.

**Results:**

Overall, 6 patients from 5 different Taiwanese families were included in our study; the patients had an identical *OSGEP* gene mutation (c.740G > A transition) and all exhibited a uniform clinical phenotype with early-onset nephrotic syndrome, craniofacial and skeletal dysmorphism, primary microcephaly with pachygyria, and death before 2 years of age. We reviewed their clinical manifestations, the prenatal and postnatal presentations and ultrasound findings, results of imaging studies, associated anomalies, and outcome on follow-up. All individuals were found to have an “aged face” comprising peculiar facial dysmorphisms. Arachnodactyly or camptodactyly were noted in all patients. Neurological findings consisted of microcephaly, hypotonia, developmental delay, and seizures. Brain imaging studies all showed pachygyria and hypomyelination. All patients developed early-onset nephrotic syndrome. The proteinuria was steroid-resistant and eventually resulted in renal function impairment. Prenatal ultrasound findings included microcephaly, intrauterine growth restriction, and oligohydramnios. Fetal MRI in 2 patients confirmed the gyral and myelin abnormalities.

**Conclusions:**

Our study suggests that a careful review of the facial features can provide useful clues for an early and accurate diagnosis. Prenatal ultrasound findings, fetal MRI, genetic counseling, and mutation analysis may be useful for an early prenatal diagnosis.

## Background

Galloway–Mowat syndrome (GAMOS) is a rare autosomal recessive disorder characterized by early-onset steroid-resistant nephrotic syndrome (SRNS) and microcephaly with brain anomalies [1]. It was originally described in 1968 in two siblings with the triad of congenital nephrotic syndrome, microcephaly, and hiatus hernia [1]. Since then, more than 60 patients have been reported in the literature and heterogeneous clinical and histopathological phenotypes were reported. Renal presentations range from asymptomatic proteinuria to SRNS. Although early-onset nephrotic syndrome is more common, later onset during childhood has also been reported. Diverse central nervous system anomalies and the involvement of multiple organs were also reported. However, the appearance of a hiatal hernia was found to be inconsistent and is no longer considered essential for the diagnosis. At present, its clinical spectrum has also expanded to include craniofacial dysmorphism, abnormality of extremities, seizure disorder, developmental delay, psychomotor retardation, hypotonia, and a variety of neuropathological findings as well as heterogeneous renal histopathology. Although some authors have tried to classify GAMOS according to the clinical presentation [2, 3], no classification is currently accepted. Recent studies have unmasked important genetic mutations in some patients with GAMOS. Homozygous mutations in *WDR73* (OMIM *251300) were first implicated in patients with GAMOS reported by Colin et al. in 2014 and later studies [4–8]. Very recently, via whole exon sequencing and high throughput exon sequencing, Braun and colleagues identified mutations in Kinase, Endopeptidase and Other Proteins of small Size (KEOPS) complex genes responsible for GAMOS (OMIM *301006, *617729, *617730, *617731) [9].

The KEOPS complex is required for a universal tRNA modification, known as the N^6^-threonyl-carbamoyl-adenosine (t^6^A) modification, which is necessary for translational accuracy and efficiency [10]. The complex is composed of 4 subunits: *LAGE3*, *OSGEP*, *TP53RK*, and *TPRKB*. A fifth member of the complex, *C14ORF142*, has been identified recently [11]. Braun et al. screened the coding regions of *LAGE3*, *OSGEP*, *TP53RK*, and *TPRKB* in 907 patients with nephrotic syndrome, including 91 individuals with GAMOS, all of which were collected through an international collaborative effort. They narrowed the group down to 37 patients with GAMOS from 32 different families with mutation in these 4 genes. Specifically, recessive *OSGEP* mutations were identified in 28 patients from 24 families, including our 5 patients with mutations of *OSGEP* gene on chromosome 14q11 [9]. The term “Galloway-Mowat syndrome 1 (GAMOS1)” is now used for GAMOS caused by mutations in *WDR73*, and “Galloway-Mowat syndrome 2-5 (GAMOS2-5)” is used for GAMOS with mutations in *LAGE3*, *OSGEP*, *TP53RK*, and *TPRKB*, respectively [9]. The purpose of this study was to present our experience in the diagnosis of GAMOS3 in Taiwan with a particular emphasis on the characteristic clinical and imaging findings.

## Methods

From January 1999 to December 2017, 6 children (3 males, 3 females) with GAMOS were diagnosed; the patients came from 5 different non-consanguineous families of Taiwanese ethnic origin. We retrospectively reviewed their medical records, extracting data on how and when the diagnosis was made, the clinical manifestations, the prenatal and postnatal presentations and ultrasound findings, results of imaging studies, associated anomalies, and outcome on follow-up. The diagnosis of “true GAMOS” was based on all of the following criteria: (1) early onset nephrotic syndrome; (2) primary microcephaly with gyral abnormalities; and (3) death in early childhood (less than 6 years). The study was approved by the Institutional Review Board of the Mackay Memorial Hospital.

The detailed method for our genetic analysis was previously described and performed by Braun et al. [9]. Whole-exome sequencing was performed with Agilent SureSelect human exome capture arrays (Thermo Fisher Scientific) with next-generation sequencing on an Illumina platform [12]. High-throughput mutation analysis was conducted using PCR-based 48.48 Access Array microfluidic technology (Fluidigm) with consecutive next-generation sequencing [13]. Using these 2 methods, the coding regions of *OSGEP*, *TP53RK*, *TPRKB*, and *LAGE3* were screened.

## Results

### Clinical characterization of the study populations

**Fig. 1:**
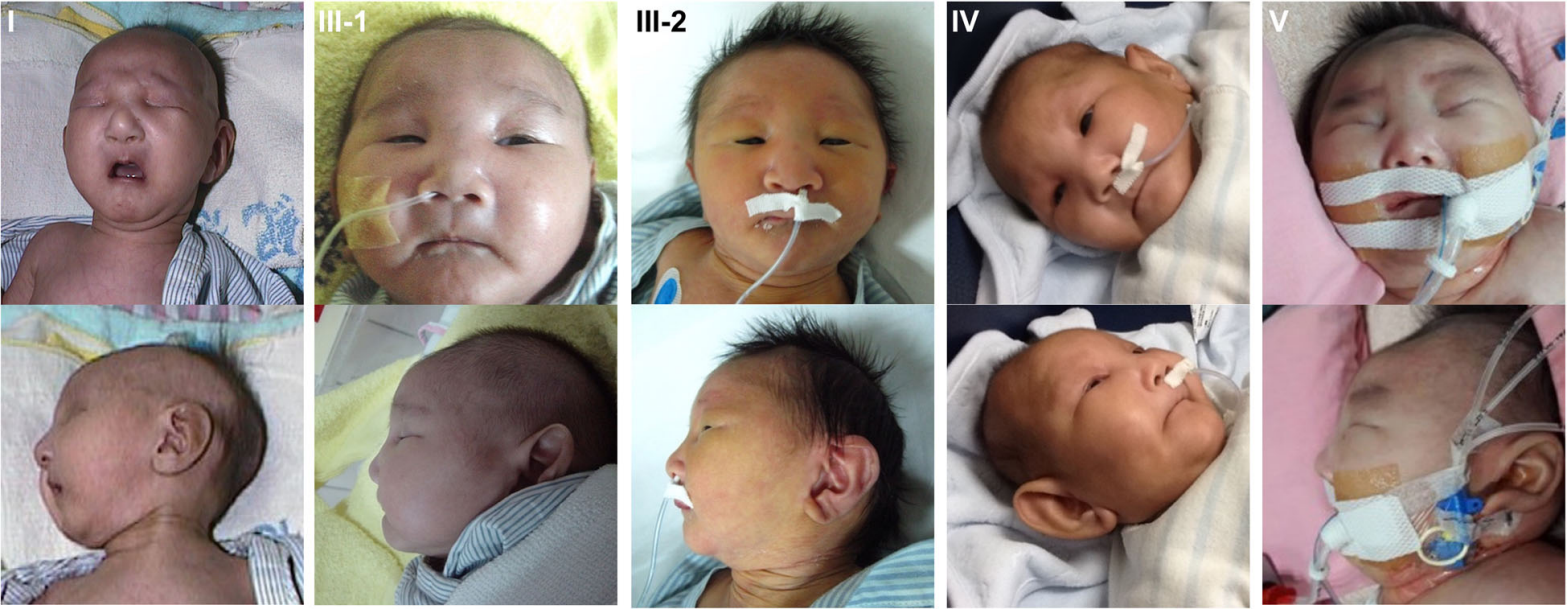
Anterior and lateral view of the patients with peculiar facial dysmorphisms, including large and floppy ears, micrognathia, hypertelorism, microphthalmia, sunken eyeballs, coarse hair, a narrow or receding forehead, a beak nose, and prominent glabella with a broad nasal bridge

**Fig. 2:**
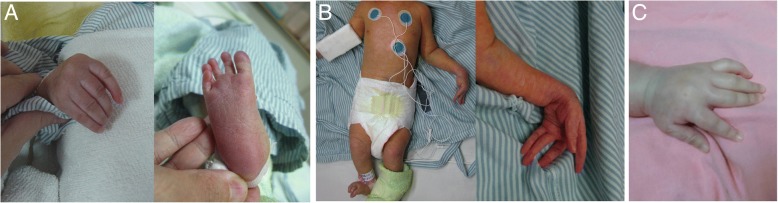
Skeletal abnormalities of the patients. **a** The hand and foot (arachnodactyly) of patient III-1. **b** Clenched hands, flexion contracture of joints, camptodactyly, and arachnodactyly of patient III-2 at birth. **c** The hand (camptodactyly) of patient V

**Fig. 3:**
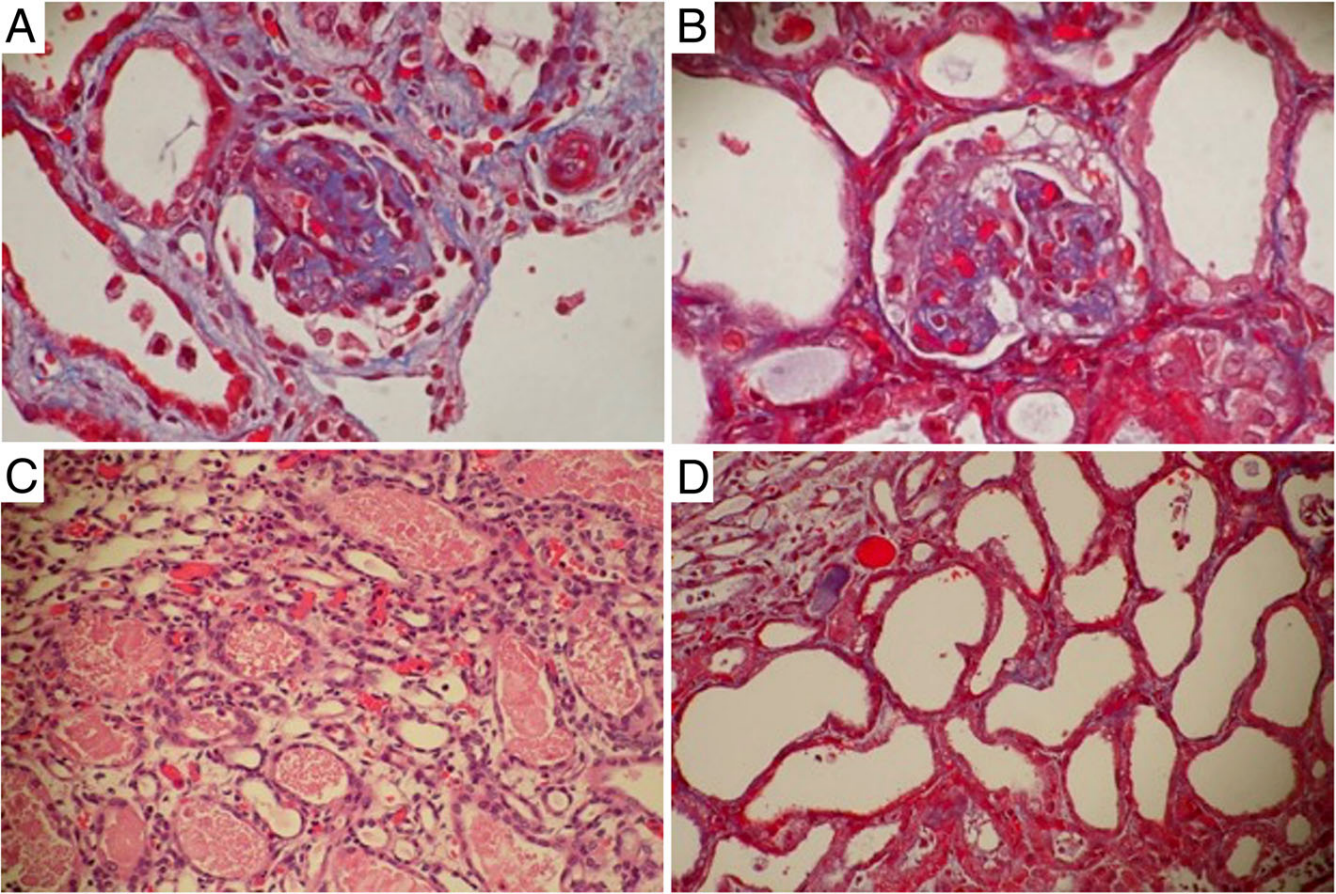
Renal pathology on light microscopy (hematoxylin-eosin stain) of patient V. **a** Glomeruli show diffuse mesangial sclerosis with increase of mesangial cells and matrix. **b** Podocyte hyperplasia is also prominent. (original magnification × 400). **c** Many granular casts are present with marked tubular ectasia. (original magnification × 200). **d** Renal tubules show intraluminal cell sloughing, vacuolization, and simplification (original magnification × 400)

Our study included 3 males and 3 females. Clinical findings are summarized in Table 1. Patients III-1 and III-2 are siblings. All of our patients were born small for gestational age (SGA) at term or near-term (Patient IV, at 36 weeks and 6 days). All individuals were found to have an “aged face” comprising peculiar facial dysmorphisms (Fig. 1). The consistent facial dysmorphic features included large and floppy ears, micrognathia, hypertelorism, microphthalmia, sunken eyeballs, coarse hair, a narrow or receding forehead, a beak nose, and prominent glabella with a broad nasal bridge. Skeletal abnormalities such as arachnodactyly or camptodactyly (Fig. 2) were also noted in all patients. Other dysmorphisms included a high arch palate (5/6). Neurological findings included microcephaly (6/6), hypotonia (6/6), developmental delay (6/6), and seizures (5/6). The earliest renal involvement was in patient II who had proteinuria on the second day after birth. All patients developed early-onset nephrotic syndrome (range, 6 days-7 weeks). The proteinuria was progressive and unresponsive to corticosteroid treatment, and this eventually resulted in massive proteinuria (range of urine protein to creatinine ratio, 20.4–740) and renal function impairment. Both Patient I and patient V had undergone a renal biopsy. In Patient I, light microscopy showed mild glomerular changes, cystic dilatation of tubular lumina, tubular atrophy with proteinaceous casts, and arteriolar medial hypertrophy (a typical picture of “microcystic disease”). Electron microscopy showed an irregular thickness of the glomerular basement membrane and complete effacement of the foot processes [14]. In Patient V, light microscopy showed diffuse mesangial sclerosis with increase of mesangial cells and matrix (Fig. 3). Electron microscopy showed global multilayering and irregular thickening of glomerular basement membrane.

**Table 1:** Clinical features of six Galloway-Mowat syndrome (GAMOS) patients

Patient	I	II	III-1	III-2	IV	V
Gender	Female	Female	Male	Female	Male	Male
GA at birth	39 weeks	38 weeks	37 weeks	40 weeks	36 weeks	38 weeks
Birth weight	2460 g (<5th percentile)	1954 g (<5th percentile)	2118 g (<5th percentile)	2496 g (<5th percentile)	2345 g (<5th percentile)	2340 g (<5th percentile)
Prenatal findings						
IUGR	+	+	+	+	+	+
Oligohydramnions (GA)	–	+ (at 38 weeks)	+ (at 32 weeks)	+ (at 27 weeks)	+ (at 34 weeks)	+ (at 35 weeks)
Microcephaly	+	+	+	+	+	+
Abnormal ultrasound findings	–	–	–	–	–	–
Fetal MRI	–	–	+	+	–	–
Neurological involvement						
Primary microcephaly	+	+	+	+	+	+
Hypotonia	+	+	+	+	+	+
Developmental delay	+	+	+	+	+	+
Seizures	+	+	+	+	–	+
Cranial imaging	MRI	CT	MRI	MRI	MRI	CT
Gyral defects (pachygyria)	+	+	+	+	+	+
Myelination defects/leukoencephalopathy	+	–	+	+	+	–
Cerebellar atrophy	–	–	–	+	–	–
Renal involvement						
Nephrotic syndrome (Onset)	6 weeks	1 month (proteinuria at 2 days)	1 month	6 days	10 days	1 month
Facial dysmorphism						
Large/floppy ears	+	+	+	+	+	+
Micrognathia	+	+	+	+	+	+
Hypertelorism	+	+	+	+	+	+
Microphthalmia	+	+	+	+	+	+
Sunken eyeballs	+	+	+	+	+	+
Narrow/receding forehead	+	+	+	+	+	+
Beak nose	+	+	+	+	+	+
Prominent glabella/ Broad nasal bridge	+	+	+	+	+	+
High arch palate	+	–	+	+	+	+
Skeletal features						
Arachnodactyly or camptodactyly	+	+	+	+	+	+
Death (Age)	3 mon	1 year 9 mon	5 mon	3 mon	3 mon	2 mon
Other abnormalities			Cryptorchidism, Micropenis, Ectopic kidney		Micropenis	Micropenis

+, Yes; −, No; *d* Days, *GA* Gestational age, *IUGR* Intrauterine growth restriction, *mon* Months

**Fig. 4:**
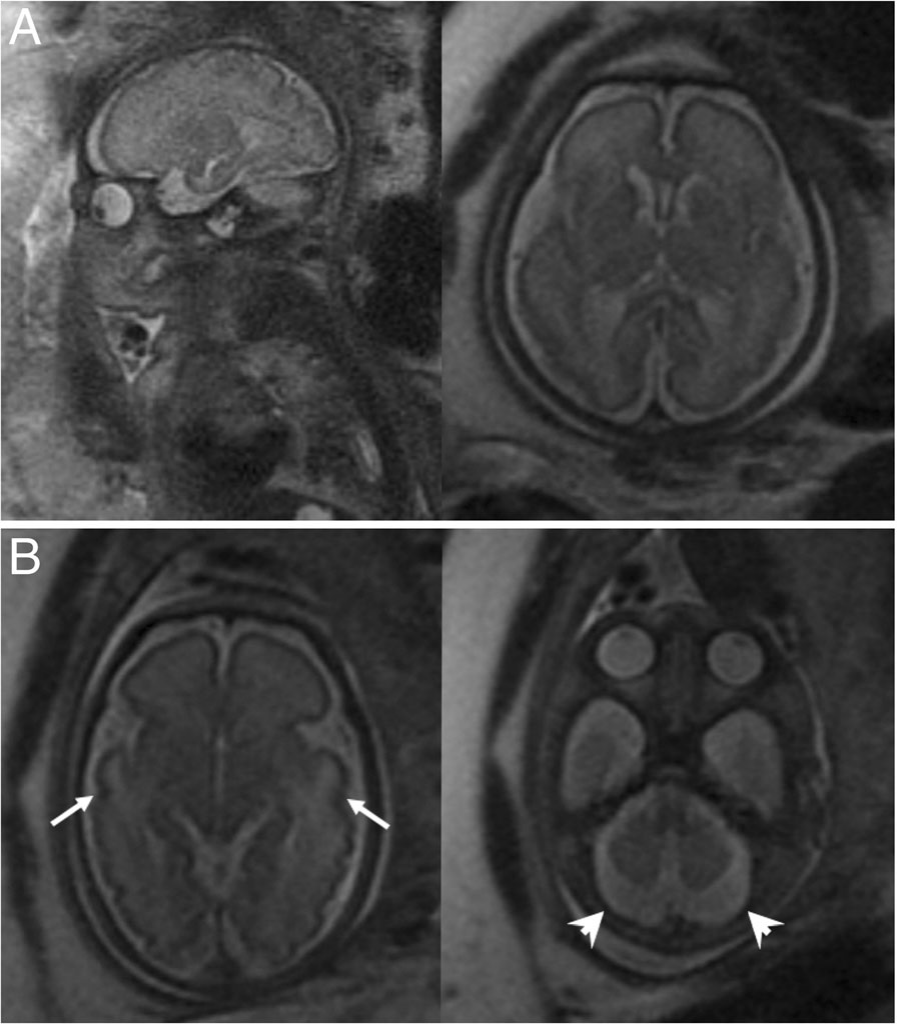
Images of fetal ultrafast MRI. **a** Patient III-1 at 34 weeks of gestation shows pachygyria, especially in the bilateral frontal lobes, and poor myelination of the white matter (**b**) Patient III-2 at 32 weeks of gestation shows hypomelination with T2 hyperintensity in bilateral cerebral white matter, particularly both temporal lobes (*arrows*), and prominence of retrocerebellar cisterns due to cerebellar atrophy (*arrowheads*)

All the prenatal ultrasound findings showed intrauterine growth retardation and microcephaly. Oligohydramnios was diagnosed in 5 out of 6 patients at 27–38 weeks of gestation. Prenatal fetal ultrafast MRI was performed in patients III-1 and patient III-2 at 34 weeks and 32 weeks of gestation, respectively (Fig. 4). In patient III-1, fetal magnetic resonance imaging (MRI) showed pachygyria, especially in the bilateral frontal lobes, and poor myelination of the white matter. In patient III-2, pachygyria was also present and an increased T2 signal of white matter, particularly in both temporal lobes, and cerebellar atrophy with an enlargement of the retrocerebellar cistern was also observed.

**Fig. 5:**
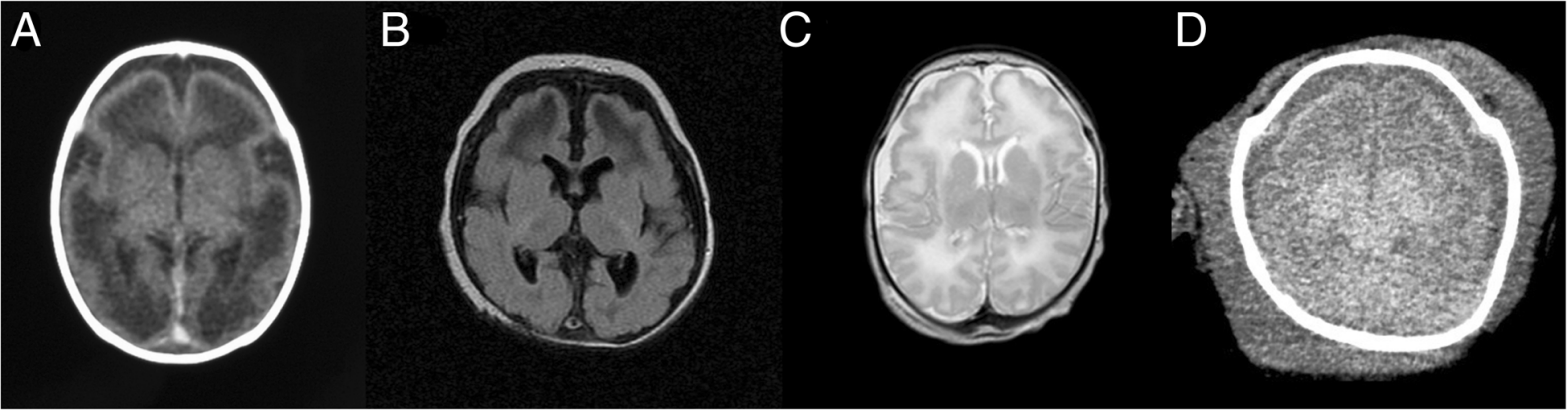
Brain images of the patients. **a** Non-contrast enhanced axial carnial CT scan of patient II at 2 days of age showed pachygyria involving bilateral cerebral hemispheres. **b** Axial sections of MRI of patient III-1 at 9 weeks of age showed gyral abnormalities, frontal pachygyria, and deficient myelination. **c** MRI of patient III-2 at 6 days of age showed pachygyria and hypomyelination with T2 high signal intensity of white matter in bilateral frontal and temporal lobes. **d** Non-contrast enhanced axial carnial CT scan of patient V at 1 month of age showed pachygyria involving bilateral cerebral hemispheres

Overall, 4 of our patients had been examined by cranial MRI after birth which revealed pachygyria and hypomyelination (Fig. 5) [15–17]. Patient II and patient V were examined by cranial computed tomography (CT) scan which showed pachygyria without a definite myelin defect. However, a lack of myelin is generally too subtle to identify on CT. Only 1 patient (patient III-2) had documented cerebellar atrophy. All patients died at early childhood because of severe proteinuria with hypoalbuminemia, deterioration of renal function, and multi-organ failure (range, 2 months–1 year 9 months).

### Molecular results

Genetic studies using whole-exome sequencing and high-throughput exon sequencing identified a homozygous mutation at c.740G > A transition (c.740G > A, NM_017807.3) in exon 8 of the *OSGEP* gene on chromosome 14q11, resulting in an arginine to glutamine substitution at codon 247 (p.R247Q), a highly conserved residue in all of the 6 subjects. The inheritance was autosomal recessive.

## Discussion

Since 1968, more than 60 cases of GAMOS have been reported with an expanding spectrum of phenotypic findings. Nephrotic syndrome with microcephaly has histologically been the major diagnostic criteria of GAMOS. These reported cases labeling GAMOS had renal involvement ranging from isolated proteinuria to early- or late-onset nephrosis, and large varieties of brain abnormalities such as primary or secondary (postnatal) microcephaly, cerebral or cerebellar atrophy, and neural migration defects [8]. Given its clinical heterogeneity, GAMOS is believed to be a genetically heterogeneous group of disorders. In this study, we report on a group of 6 patients with an identical *OSGEP* gene mutation (c.740G > A transition) who exhibited a uniform clinical phenotype with early-onset SRNS, craniofacial and skeletal dysmorphism, primary microcephaly with cerebral pachygyria, and early death before 2 years of age. In the study by Braun et al., with only one exception, 9 out of 10 GAMOS patients of Taiwanese ethnic origin were found to have a c.740G > A transition in the *OSGEP* gene [9]. On the basis of a shared the same *OSGEP* gene mutation and geographical location, preliminary evidence suggested the presence of a founder effect among GAMOS cases in the Taiwan population. The frequency of reports of GAMOS from Taiwan is striking considering the extreme rareness of this syndrome [14–17]. This is very likely due to a high allele frequency of the founder mutation that has been demonstrated in almost all Taiwanese cases. The Exome Aggregation Consortium (ExAC; http://exac.broadinstitute.org) gives an allele frequency for this mutation of 0.0008 in the East Asian population [18]. This would anticipate an incidence of the disease of about 1 in a million in this population. The frequency in subpopulations of East Asia may be even higher.

Our cases are consistent with the group of patients with true GAMOS (microcephaly, gyral abnormalities, and early-onset nephrotic syndrome) proposed by Meyers and Keith [3, 19]. Meyers et al. suggested that the term GAMOS should be reserved for patients with early-onset nephrotic syndrome, microcephaly with gyral abnormalities, and death in early childhood [3]. From a pathological point of view, Keith et al. showed that children with disordered neuronal migration tend to have a worse prognosis (true GAMOS), and those without gyral abnormalities have a better prognosis [19].

Although previous studies had documented the facial dysmorphic features as minor and not specific to this syndrome [3, 14], all of our patients were born with an aged face with the features such as large and floppy ears, micrognathia, hypertelorism, microphthalmia, sunken eyeballs, coarse hair, a narrow or receding forehead, a beak nose, and prominent glabella with a broad nasal bridge. These dysmorphic facies can be an important factor in the diagnosis of GAMOS. For our patients II and III-1, with these typical characteristic facies after birth, GAMOS was highly suspected and signaled the need to verify the existence of proteinuria. Edema and nephrotic syndrome then developed later during a subsequent follow-up. Arachnodactyly or camptodactyly was noted in all of our patients (caused by an *OSGEP* mutation) and has been frequently observed in Taiwanese patients affected with GAMOS [9, 14–17]. This geographic difference may be due to our special founder mutation in the *OSGEP* gene (c.740G > A transition) specific to Taiwanese patients. It is also possible that it was present in the other reported GAMOS patients but not included in the published papers either because it went unnoticed or was not considered a distinctive enough feature worth mentioning.

Typical brain MRI findings for true GAMOS include a spectrum of gyration abnormalities ranging from lissencephaly to pachygyria and polymicrogyria, myelination defect, and cerebellar hypoplasia. The clinical neurologic manifestations in our patients included microcephaly at birth (6/6), global developmental delay (6/6), hypotonia (6/6), intractable seizure (5/6), and structural brain abnormalities, including pachygyria (6/6), myelination defect (4/6), and cerebellar hypoplasia (1/6). In our country, all pregnant women are permitted a routine prenatal ultrasound that is covered by the National Health Insurance. Although the kidneys are unremarkable at the fetal stage, the prenatal significant sonographic triad includes microcephaly (6/6), intrauterine growth retardation (6/6), and oligohydramnios in the second or third trimester (5/6). It implies that the neurological and growth abnormalities are universal in children with GAMOS3, often precede renal symptoms [20], and even begin during the prenatal stage. Fetal ultrafast MRI can be used for prenatal diagnosis and assessment of fetal sulci, gyral abnormalities, and cerebellar atrophy, as in 2 of our patients. An in vivo, knockout of orthologous KEOPS subunit genes in zebrafish and mouse recapitulated the primary microcephaly phenotype but not the renal phenotype seen in GAMOS patients [9]. The early lethality in animal models could have masked the renal presentation that might have been seen in older animals.

Concerning the renal involvement, all our patients developed early-onset (6 days-7 weeks) nephrotic syndrome. Previously reported glomerular findings on a light microscope were inconsistent and may have varied from minimal change disease, focal segmental glomerulosclerosis, diffuse mesangial sclerosis, collapsing glomerulopathy, or microcystic dysplasia [14, 20] and were also described in patients with GAMOS3 [9]. The inconsistencies may be due to evaluation of patients at different ages or stages of the disorder [14]. Using an electron microscope, our previous study suggested the pathognomonic pathological features of “true GAMOS” were irregular thickness of the glomerular basement membranes and the effacement of foot processes [14]. An in vivo study recently showed that knockdown of *OSGEP* and *TP53RK* resulted in defects in the actin cytoskeleton and a decrease of human podocyte migration rate [9]; these findings are compatible with the pathological manifestation in the development of nephrotic syndrome [9]. The prognosis for patients with GAMOS3 is poor. All of our patients had SRNS, followed shortly by end-stage renal disease and early death.

The N^6^-threonyl-carbamoyl-adenosine (t^6^A) modification, one of the numerous post-transcriptional tRNA modifications, is a complex modification of adenosine located at position 37 (t^6^A37) next to the anticodon stem loop of many tRNAs that decode ANN codons. The absence of this modification is associated with a severe growth phenotype in yeast [21]. Edvardson et al. postulated that *OSGEP* mutation exerts its pathogenic effect by perturbing t^6^A synthesis, thereby interfering with global protein production, which leads to neurodegeneration and renal tubulopathy [22]. By knockdown of *OSGEP* using shRNA in human podocytes in vitro, Braun et al. further proved that *OSGEP* mutations impair the functions of the KEOPS complex, resulting in disturbed translation, endoplasmic reticulum stress, dysfunctional DNA damage response, disturbance of actin regulation, and ultimately apoptosis [9].

The features of our patients are different from patients with *WDR73*-positive GAMOS (GAMOS1). Table 2 lists the different presentations of patients with GAMOS3 and GAMOS1. *WDR73* plays an important role in the maintenance of cell architecture and cell survival [4]. Patients with *WDR73*-positive GAMOS are rarely associated with the typical GAMOS phenotype, but rather present with a predominantly infantile-onset neurodegenerative disease with variable renal involvement [7]. Mutations of *WDR73* are only found in a small subset (2/31 and 2/40) of patients with GMOS [4, 7]. The main clinical manifestations included postnatal microcephaly, a coarse face, severe intellectual disability, seizures, cerebellar ataxia, optic atrophy, and late-onset nephrotic syndrome [4–8]. Kidney involvement typically occurs in later years (2–8 years of age, median age, 5) with mostly slow progressing nephrotic syndrome [4–8]. The most consistent finding on brain MRI is cerebellar atrophy. Cerebellar atrophy is present for all GAMOS1 patients reported so far and is considered a notable predictive feature for diagnosis of the *WDR73* mutation [4–7]. Cerebellar atrophy was also found in 9 out of 28 patients with *OSGEP* mutations [9], including our patient III-2. However, gyral abnormalities and myelin defect were not present in patients with *WDR73*-positive GAMOS. These differences are important for diagnostic, therapeutic, prognostic, and genetic counseling purposes.

**Table 2:** Different presentations between patients with GAMOS3 and GAMOS1

Characteristics	GAMOS3	GAMOS1
Mutant gene	OSGEP on chromosome 14q11	WDR73 on chromosome 15q25
Pattern of inheritance	Autosomal recessive	Autosomal recessive
Frequency in patients with GAMOS	High (24 of 32 families) [9]	Low (2 of 31 or 2 of 40 families) [4, 7]
Facial dysmorphism	The aged face comprising features: Narrow forehead, large floppy ears, deep-set eyes, coarse hair, beaked nose, prominent nasal bridge, microphthalmia, hypertelorism, micrognathia, high-arch palate [9, 14–17]	Coarse facial features: full lips and cheeks, broad forehead, bushy eyebrows, flat nasal root with fleshy nasal tip [8]
Skeletal deformity	Arachnodactyly, camptodactyly, clenched hands	No
Renal features		
Age of onset of proteinuria	Early-onset (< 3 months)	Late-onset
Degree of renal disease	Heavy proteinuria with Nephrotic syndrome	Variability, from no renal involvement, mild proteinuria, to nephrotic syndrome
Renal lesions	Light microscope: minimal change disease, focal segmental glomerulosclerosis (FSGS), diffuse mesangial sclerosis, collapsing glomerulopathy, or microcystic dysplasia [9]	Light microscope: FSGS, collapsing FSGS [4, 8]
	Electron microscope: foot process effacement, irregular thickness of the glomerular basement membranes [9, 14]	Electron microscope: podocyte hypertrophy [4, 8]
End stage renal disease	Common	Rare
Neurological features		
Microcephaly	Primary (prenatal) microcephaly	Secondary (postnatal) microcephaly
Clinical presentations	Severe psychomotor retardation, global developmental delay, hypotonia, intractable seizure	Neurodegenerative course, developmental delay, seizure, intellectual disability, cerebellar ataxia, optic atrophy [4–8]
Brain MRI	Gyral abnormalities (all): lissencephaly, pachygyria, or polymicrogyria Myelination defect (most) Cerebellar atrophy (some, 9 of 28) [9]	Cerebellar atrophy (all) [4–8] Other abnormalities: thin corpus callosum, brain stem hypoplasia [4–8]
Prenatal ultrasonographic findings	Microcephaly, intrauterine growth retardation, and oligohydramnios	No specific findings

## Conclusions

We present a typical and specific group of GAMOS patients with consistent clinical phenotype and identical genetic mutations, the *OSGEP* mutation in Taiwan. Our patients had a highly concordant clinical phenotype of GAMOS comprising facial and extremity dysmorphism, early-onset SRNS, primary microcephaly with abnormal gyri and migration anomalies, severe developmental delay, a propensity for seizure, and death in early childhood. The aged face and arachnodactyly or camptodactyly are obvious at birth. A careful study of the facial features can provide useful clues for the early and accurate diagnosis of GAMOS3. Prenatal ultrasound findings include microcephaly, intrauterine growth restriction, and oligohydramnios. For a suspected case, fetal MRI may be useful for a detailed search of gyral maldevelopment, myelin defect, and other brain anomalies. Genetic counseling and mutation analysis should be part of the standard care for these patients.

## Abbrevitations

CT: Computed tomography
ExAC: Exome Aggregation Consortium
GAMOS: Galloway-Mowat syndrome
KEOPS: Kinase, Endopeptidase and Other Proteins of small Size
MRI: Magnetic resonance imaging
SGA: Small for gestational age
SRNS: Steroid-resistant nephrotic syndrome
t^6^A: N^6^-threonyl-carbamoyl-adenosine
